# Potential dual imaging nanoparticle: Gd_2_O_3_ nanoparticle

**DOI:** 10.1038/srep08549

**Published:** 2015-02-24

**Authors:** Md. Wasi Ahmad, Wenlong Xu, Sung June Kim, Jong Su Baeck, Yongmin Chang, Ji Eun Bae, Kwon Seok Chae, Ji Ae Park, Tae Jeong Kim, Gang Ho Lee

**Affiliations:** 1 Department of Chemistry, College of Natural Sciences, Kyungpook National University (KNU), Taegu 702-701, South Korea; 2 Department of Molecular Medicine and Medical & Biological Engineering, School of Medicine, KNU, Taegu 702-701, South Korea; 3 Department of Nanoscience and Nanotechnology, KNU, Taegu 702-701, South Korea; 4 Department of Biology Education, Teacher's College, KNU, Taegu 702-701, South Korea; 5 Laboratory of Nuclear Medicine Research, Molecular Imaging Research Center, Korea Institute of Radiological Medical Science, Nowon-gil 75, Seoul 139-706, South Korea; 6 Institute of Biomedical Engineering Research, Kyungpook National University, Taegu 702-701, South Korea

## Abstract

Gadolinium (Gd) is a unique and powerful element in chemistry and biomedicine which can be applied simultaneously to magnetic resonance imaging (MRI), X-ray computed tomography (CT), and neutron capture therapy for cancers. This multifunctionality can be maximized using gadolinium oxide (Gd_2_O_3_) nanoparticles (GNPs) because of the large amount of Gd per GNP, making both diagnosis and therapy (i.e., theragnosis) for cancers possible using only GNPs. In this study, the T_1_ MRI and CT dual imaging capability of GNPs is explored by synthesizing various iodine compound (IC) coated GNPs (IC-GNPs). All the IC-GNP samples showed stronger X-ray absorption and larger longitudinal water proton relaxivities (r_1_ = 26–38 s^−1^mM^−1^ and r_2_/r_1_ = 1.4–1.9) than the respective commercial contrast agents. In vivo T_1_ MR and CT images of mice were also acquired, supporting that the GNP is a potential dual imaging agent.

During the last decade, various nanoparticles have been introduced into biology and medicine^1^,^2^,^3^,^4^,^5^,^6^,^7^,^8^,^9^,^10^,^11^,^12^ because of their advanced physical and chemical properties which are better than those of small molecules^9^,^10^,^11^,^12^,^13^,^14^,^15^,^16^,^17^. In addition, nanoparticles can allow easy surface modifications for targeting and drug delivery^18^,^19^,^20^,^21^,^22^,^23^ and longer blood circulation times than small molecules^24^,^25^,^26^, providing more imaging time and more possibilities for targeting and drug delivery to desired sites such as cancers.

Among nanoparticles, the gadolinium oxide (Gd_2_O_3_) nanoparticle (GNP) seems to be special because of its diagnostic and therapeutic properties (Fig. 1)^11^,^27^,^28^,^29^. This multifunctionality arises from the high spin magnetic moment (s = 7/2) of a trivalent Gd(III) (^8^S_7/2_) (the *largest* value among the elements in the periodic table), which is useful for magnetic resonance imaging (MRI)^30^,^31^; the very high X-ray attenuation (or absorption) coefficient of Gd [less than that of gold but higher than that of iodine (I) that is commercially used as a CT contrast agent in triiodinic molecular forms], which is useful for X-ray computed tomography (CT)^32^,^33^,^34^,^35^; and the huge thermal neutron capture cross-section of ^157^Gd (15.6% natural abundance) of 257,000 barns (the *largest* value among the known stable radio-isotopes), which is useful for neutron capture therapy (NCT) for cancers^36^,^37^. Therefore, both diagnosis and therapy (i.e., theragnosis) for cancers will be possible using only GNPs using these remarkable properties of Gd. In addition, GNPs will have advantages over complex nanoparticles such as core-shell^38^, hetero-junction^39^, or hybrid nanoparticles^40^ because of its simple synthesis, compactness, and robustness.

In this study, the dual imaging capability of GNPs in T_1_ MRI and CT is explored in vitro and in vivo. MRI and CT are the most commonly used imaging modalities in clinical trials, primarily because they can provide three-dimensional tomographic information on the body^41^,^42^,^43^,^44^,^45^. Using contrast agents, however, can further improve both the resolution and sensitivity^30^,^31^,^46^,^47^,^48^,^49^,^50^. Currently used commercial contrast agents in these imaging modalities include Gd(III)-diethylenetriamine pentaacetic acid (Gd-DTPA) and Gd(III)-1,4,7,10-tetraazacyclododecane-1,4,7,10-tetraacetic acid (Gd-DOTA) for T_1_ MRI^3^,^4^, and iodine compounds for CT^46^,^47^,^48^. Because Gd has a higher X-ray attenuation coefficient than iodine^35^, Gd-DTPA had been examined as a CT contrast agent^33^,^51^,^52^,^53^,^54^. However, Gd-chelates are not used as CT contrast agents because they can be concentrated up to only 0.5 M Gd because there is only one Gd per molecule, whereas iodine contrast agents can be highly concentrated, up to 1.0–2.5 M I, because there are three iodines per monomer-molecule and six iodines per dimer-molecule^51^. To provide adequate contrast, however, large doses of iodine contrast agents are generally administered, which might cause potential side-effects in patients^55^. However, the injection doses can be reduced using nanoparticles because at the same atomic concentration, the number density of nanoparticles is much lower than those of molecular agents^26^. Furthermore, because of Gd's higher X-ray attenuation coefficient^35^, the injection doses of GNPs can be further reduced.

The diagnostic and therapeutic applications of GNPs are summarized in Fig. 1. Although not examined in this study, GNPs can also be applied to NCT for cancers (Fig. 1). Note that NCT has been proved to be powerful for noninvasively treating malignant brain cancers^56^,^57^,^58^,^59^. Two ^10^B-chemicals (B = boron) have been developed for clinical purpose^59^. However, ^157^Gd, with a ~67 times higher thermal neutron capture cross section than ^10^B (19.97% natural abundance), is expected to be even more powerful than ^10^B-NCT^60^,^61^,^62^. This, when combined with the imaging property of GNPs, will make GNPs a powerful theragnostic agent for cancers^11^,^27^,^28^,^29^, whereas ^10^B-chemicals are useful only as therapeutic agents for cancers because they have no diagnostic capability.

This study examines the dual imaging capability of GNPs. To this end, various iodine compound (IC) coated GNPs (IC-GNPs) were synthesized, and their T_1_ MRI and CT functionalities were investigated in vitro and in vivo. Four commercial iodine contrast agents were used as surface coating ligands on GNPs to maximize the CT functionality of the nanoparticles because of the iodines in the ICs, as well as to make them water-soluble and biocompatible. To prove the potential of GNPs as a dual imaging agent, the water proton relaxivities, X-ray absorption, and in vitro cellular cytotoxicities were measured, and finally in vivo T_1_ MR and CT images of mice were acquired after intravenous injection.

## Results

### Particle diameter and hydrodynamic diameter

As shown in high-resolution transmission electron microscope (HRTEM) images (Fig. 2a), the diameters of the nanoparticles range from 1 to 3 nm for all the samples. Arrows indicate individual nanoparticles. Gd and I in Sample 2 were mapped onto a high-angle annular dark field - scanning transmission electron microscopy (HAADF-STEM) image (Fig. 2b) to see the Gd and I distributions in the nanoparticle. As expected, a dense Gd population at the nanoparticle core and widely spread I over the nanoparticle were observed. The average particle diameters were estimated from log-normal function fits to the observed particle diameter distributions (Fig. 2c and Table 1). The average hydrodynamic particle diameters were also estimated from log-normal function fits to the observed dynamic light scattering (DLS) patterns (Fig. 2d and Table 1). Very broad X-ray diffraction (XRD) patterns were observed for all the powder samples, likely owing to the ultrasmall particle diameters^63^, whereas sharp peaks corresponding to cubic Gd_2_O_3_, were observed for all the samples after thermo-gravimetric analysis (TGA) (Supplementary Information). This is due to particle growth during TGA treatment^63^,^64^. The estimated cell constant of the TGA treated samples was 10.81 Å, which is consistent with the literature value for cubic Gd_2_O_3_ ( = 10.813 Å)^65^.

We carried out the long-term colloidal stability study in physiological conditions (i.e., pH = 7.0, 1 mM glucose, 1 atm, and room temperature), and we found that the colloidal stability maintained for a week for all sample solutions. After IC-GNPs settled down, however, they could be readily re-dispersed again as stable colloids in solutions. Photographs of 1 mM Gd sample solutions showing stable colloidal dispersions in physiological conditions are provided in Fig. 2e. We also estimated the free Gd^3+^ ion concentrations liberated from IC-GNPs in aqueous sample solutions with 1.0 mM Gd, but the free Gd^3+^ concentrations were below the detection limit of the ICP-AES (i.e., <0.1 ppm Gd) in all sample solutions for one month.

### Surface coating results

Surface coating of GNPs with ICs was investigated by recording Fourier transform infrared (FT-IR) absorption spectra (Fig. 3a). The absorption peaks from ICs in the samples showed that the GNPs were successfully coated with ICs in all the samples. Overall, the absorption peaks of all the samples were broad and overlapped with neighboring peaks. The assignments of some important peaks are provided in the Supplementary Information.

A sufficient surface coating of GNPs with ICs is crucial because both water-solubility and biocompatibility are necessary for biomedical applications. For example, Gd may cause nephrogenic systemic fibrosis when it is released in the body during circulation^66^. The amount of surface coating on each sample was estimated in weight percent (%) ( = P) from the mass drop in its TGA curve (Fig. 3b). Here, an initial mass drop due to moisture desorption between room temperature and ~105°C was subtracted in estimating P. Then, the grafting density ( = σ)^67^, which corresponds to the average number of ICs coated per unit surface area of a GNP, was estimated using the bulk density of Gd_2_O_3_ ( = 7.407 g/cm^3^)^68^, P, and the average particle diameter ( = d_avg_). By multiplying the estimated σ by the nanoparticle surface area ( = πd_avg_^2^), the average number ( = N) of ICs coated per GNP was estimated using N = σπd_avg_^2^. Finally, the average number ( = NI) of iodines per GNP was estimated by multiplying N by the number of iodines per IC (i.e., NI = 3N for Samples 1 and 3, and 6N for Samples 2 and 4). The estimated P, σ, N, and NI values are provided in Table 1 and plotted as a function of the IC mass in Fig. 3c. ICs with a larger mass had smaller P, σ, N, and NI values, indicating that fewer molecules of an IC with a larger mass were coated on each GNP. This is because a more massive IC occupies a greater volume on the GNP surface.

An IC is bonded to a GNP through its functional group, such as COOH, NH_2_, or OH. This bonding corresponds to a hard acid [Gd(III)]-hard base (functional group of IC) type of reaction^69^,^70^,^71^. The bonding strength of these functional groups to the GNP is likely to be in the order COOH > NH_2_ > OH in triethylene glycol solvent. According to the FT-IR absorption spectra, the initially coated triethylene glycol on the GNPs was replaced by the ICs used in this study, indicating that the OH is the weakest bonding group among the above three functional groups. The strongly bonded COOH generally shows a red-shift with respect to free COOH, as observed in many cases^63^,^72^,^73^,^74^,^75^. The red-shift in this study was observed to be 74–75 cm^−1^ in Samples 1, 2 and 3, as indicated by arrows in Figs. 3a(1) to 3a(3). Among the two functional groups COOH and NH_2_ in Sample 1, the more strongly bonding COOH group was bonded to the GNPs, as indicated by the red shift of the bonded COOH in Sample 1 [Fig. 3a(1)]. The IC in Sample 4 has many OH groups, so it is likely that any OH groups that are geometrically accessible to the GNP can be bonded to it. This is likely an entropy-driven replacement reaction of the initially coated triethylene glycol on GNPs with iodixanol, which has many OH groups. Because of the geometrical difficulty of both COOH groups in Samples 1 and 2 accessing the GNP, only one of them is likely bonded to the GNP. Figure 4 shows the most probable bonding structures between the ICs and GNPs in the four samples according to these results.

The elemental analyses of surface coated ICs on GNPs were also studied using both the elemental analyzer (EA) and the X-ray photoelectron spectrometer (XPS). The EA (C, H, O, N) elemental analyses (Supplementary Information) show that the total weight percents (i.e., P) are 45.2, 38.0, 55.1, and 37.2 for samples 1, 2, 3, and 4, respectively. These values are roughly consistent with the respective P values estimated from TGA curves, as shown in Fig. 3d. The differences in P between TGA and EA data are likely owing to moisture because the moisture content of EA data could not be eliminated, whereas it was eliminated in TGA data (Fig. 3b). The XPS spectra also clearly showed the fingerprint transitions of iodine [i.e., 618.8 eV (3d_5/2_) and 630.2 eV (3d_3/2_) in electron binding energy (EBE) scale] in all samples (Fig. 3e), confirming the surface coated ICs on GNP surfaces in all samples. The full scan XPS spectra with transition assignments in all samples are provided in Supplementary Information.

### Magnetic properties

The mass-corrected magnetization (M) (emu/g) versus the applied field (H) (i.e., M - H) curves (−5 ≤ H ≤ 5 tesla) at a temperature T of 5 or 300 K, and the mass-corrected zero-field-cooled (ZFC) M versus T (i.e., M - T) curves (5 ≤ T ≤ 300 K) at H = 0 Oe are shown in Figs. 5. The M - H curves at T = 5 and 300 K (Fig. 5a) show that both the coercivity and the remanence are zero in all the samples (i.e., no hysteresis occurs). This lack of hysteresis and the absence of a magnetic transition down to T = 5 K in the M - T curves (Fig. 5b) show that all the samples are paramagnetic down to 5 K, like bulk Gd_2_O_3_^76^,^77^,^78^,^79^. From the M - H curves, the net M values of the GNPs were estimated for all the samples (Table 1). These unsaturated net M values of the GNPs at 5 K are even larger than the saturation M values of ferrites (MnFe_2_O_4_ = 80 emu/g, Fe_3_O_4_ = 92 emu/g, CoFe_2_O_4_ = 80 emu/g)^80^ because of the seven unpaired 4f-electrons in Gd(III). The net M values of the GNPs at 300 K are also appreciable. These appreciable M values of GNPs at room temperature and the dense population of Gd(III) in the GNPs are responsible for the larger r_1_ values of the IC-GNP samples compared to conventional Gd-chelates. The magnetization values in Table 1 are lower than those (i.e., 190–200 emu/g)^64^ of the previous measurements. These are owing to the overestimated net Gd_2_O_3_ masses from TGA curves because iodines were not completely removed from the samples during TGA analyses. This was confirmed from iodine signals in XPS spectra of the TGA analyzed samples (Supplementary Information). This is likely because solid iodine compounds with either oxygen or gadolinium were formed during TGA analyses.

### Relaxometric and X-ray absorption results

The longitudinal water proton relaxivity (r_1_) should be large, and the r_2_/r_1_ ratio should be close to one (where r_2_ is the transverse water proton relaxivity) for T_1_ MRI contrast agents because the r_2_ value is theoretically always greater than the r_1_ value^30^,^31^,^41^, and the X-ray absorption should be strong for CT contrast agents. As discussed below, GNPs satisfy all these conditions.

The r_1_ and r_2_ values of aqueous sample solutions were estimated from the slopes of plots of the inverse longitudinal (T_1_) and transverse (T_2_) relaxation times (i.e., 1/T_1_ and 1/T_2_), respectively, as a function of the Gd concentration (Fig. 6a). The estimated r_1_ and r_2_ values of all the samples (Table 2) were larger than those of Gd-chelates, and their r_2_/r_1_ ratios were also close to one (i.e., between 1.0 and 2.0). All the sample solutions also showed clear dose-dependent contrast enhancements in their longitudinal (R_1_) and transverse (R_2_) map images (Fig. 6b). These results show that all the samples are potential T_1_ MRI contrast agents with better relaxometric properties than commercial contrast agents. The differences in r_1_ and r_2_ values between samples can be attributed to the ICs used for surface coating because all the samples have similar GNP diameters. The water-accessibility to the core GNP depends on the ligand-size^81^. In general, fewer water molecules can access the core GNP for a large ligand coating, reducing the r_1_ and r_2_ values^81^. The observed r_1_ and r_2_ values showed this trend (Fig. 6c and Table 2), supporting the suggestion that the differences in r_1_ and r_2_ values between samples are due to the ICs.

Next, the X-ray absorption at an X-ray source voltage of 70 kV is plotted as a function of the Gd (or I) concentration (Fig. 6d); the observed X-ray absorption of all the samples is stronger than those of commercial contrast agents Omniscan and Ultravist because both Gd and I absorb X-ray radiation in the samples, whereas only I absorbs X-ray radiation in Ultravist and only Gd absorbs X-ray radiation in Omniscan. Here, water is a reference with 0.0 Hounsfield units (HU). X-ray absorption phantom images acquired at 100 mM Gd (or I) are also shown in Fig. 6d; the phantom images of all the samples are brighter than those of Omniscan and Ultravist. These results show that all the samples are potential CT contrast agents with X-ray absorption values higher than those of commercial contrast agents. More X-ray absorption phantom images are provided in the Supplementary Information. The observed X-ray absorption powers are in the order Sample 1 > Sample 2 ≈ Sample 4. This is because the core GNP is the same for all the samples and because the number (NI) of iodines per GNP is in the same order: NI (Sample 1) > NI (Sample 2) ≈ NI (Sample 4) (Table 1). Here, the X-ray absorption of Sample 3 was not measured owing to its low concentration, but it is expected to be comparable to that of Sample 1 because NI (Sample 1) ≈ NI (Sample 3) (Table 1).

### In vitro cytotoxicity results

Human prostate cancer (DU145) and normal mouse hepatocyte (NCTC1469) cell lines were used as test cells. The cells were incubated with IC-GNP samples for 48 hours. The cell viabilities of all the samples were good for a tested Gd concentration range of up to 500 μM (Figs. 7a and b). Therefore, all the samples are biocompatible. This is because all the ICs used are commercial CT contrast agents and because all the samples are sufficiently coated with ICs, as shown by the TGA data (Fig. 3b).

### In vivo results: T_1_ MR and CT images

Sample 1, with the largest r_1_ value among the four samples, was used to acquire in vivo T_1_ MR images of mice from the Institute of Cancer Research (ICR), USA at an MR field of 1.5 tesla. Approximately 0.1 mmol Gd/kg was injected into a mouse tail vein, and T_1_ MR images were acquired before and after injection (Figs. 8a and b). Appreciably positive (or brighter) contrast enhancements were observed in the mouse liver (labeled L), kidneys (labeled K), and aorta (labeled A) after injection, but returned almost to the initial contrast (i.e., the contrast before injection) in the liver 90 minutes after injection (Fig. 8a), and in the kidneys and aorta 15 minutes after injection (Fig. 8b), because nanoparticles were excreted from the respective organs. The signal to noise ratios (SNRs) of the regions of interest (ROIs) in the liver and in the cortex and medulla of the kidney (indicated with small dotted circles) are plotted as a function of the time after injection (Fig. 8c), showing a decrease in the SNR in both the liver and kidney with time as a result of excretion of nanoparticles from the respective organs. These results show that the sample solution is a potential T_1_ MRI contrast agent. More T_1_ MR images of Sample 1 are provided in the Supplementary Information.

Sample 1, with the largest X-ray absorption among the four samples, was also used to acquire in vivo CT images at an X-ray source voltage of 70 kV. Approximately 0.53 mmol Gd/kg was injected into an ICR mouse tail vein, and in vivo CT images were acquired before and after injection (Fig. 8d). This injection dose is much smaller than 2–6.4 mmol I/kg, which was used for iodine contrast agents in an ICR mouse^82^. Brighter contrast enhancements were observed in the mouse bladder (labeled B) after injection, and they maintained up to more than 210 minutes after injection. The X-ray absorption of the ROI in the bladder (indicated with a small dotted circle) was plotted as a function of the time (Fig. 8e); the contrast reached the maximum value at ~30 minutes after injection and then decreased, indicating that the sample solution was excreted through the bladder as urine. This result shows that the sample solution is a potential CT contrast agent. A similar result was obtained for Sample 2 and is provided in the Supplementary Information.

We performed additional in vivo CT and MR imaging experiments to compare one of our samples (i.e., Sample 4) with commercial MRI (i.e., Omniscan) and CT (i.e., Ultravist) contrast agents at the same injection doses. In vivo MR coronal images of a rat obtained with the Sample 4 and Omniscan at 12 hours after intravenous injection into its tail are shown in Fig. 9a(i) and (ii), respectively. Compared to Omniscan, the Sample 4 showed stronger signal enhancements in the liver. This can be clearly seen in the mean signal to noise ratio (SNR) plot in Fig. 9a(iii). In a similar way, in vivo CT axial images of a rat obtained with the Sample 4 and Ultravist at 3 minutes after intravenous injection into its tail are shown in Fig. 9b(i) and (ii), respectively. Compared to Ultravist, the Sample 4 showed stronger signal enhancements in the liver. This can be clearly seen in the mean signal intensity plot in Fig. 9b(iii). The injection doses were ~0.64 mmol Gd/kg for MR image measurements and ~0.53 mmol Gd/kg for CT image measurements. Sprague Dawley (SD) rats (4 weeks, male) were used.

### Comparison between samples

Based on r_1_ values and X-ray absorption powers, the in vitro T_1_ MRI and CT capability evaluations of all the samples are summarized in Fig. 10. As shown in Fig. 10, it seems that the combined capabilities of T_1_ MRI and CT are roughly in the order of Sample 1 ≈ Sample 3 > Sample 2 ≈ Sample 4. Here, the X-ray absorption of Sample 3 (slashed column) is drawn according to the expectation that it will be comparable to that of Sample 1 because the core is the same GNP and because NI (Sample 1) ≈ NI (Sample 3) (Table 1). Overall, GNPs with a smaller (i.e., less massive) IC coating showed larger r_1_ values and higher X-ray absorption. This is because more water molecules can access the core GNP for a smaller IC coating, providing the larger r_1_ values^81^, and because GNPs with a smaller IC coating had larger NI values (Table 1), giving higher X-ray absorption, which explains the observed r_1_ values and X-ray absorption results in Fig. 10. However, all the samples showed larger r_1_ values than commercial T_1_ MRI contrast agents because of the GNPs as they contained, and higher X-ray absorption than commercial iodine contrast agents because of both the Gd and I as they contained. Therefore, all the samples should be potential dual imaging agents in T_1_ MRI and CT.

## Discussion

**Figure 1:**
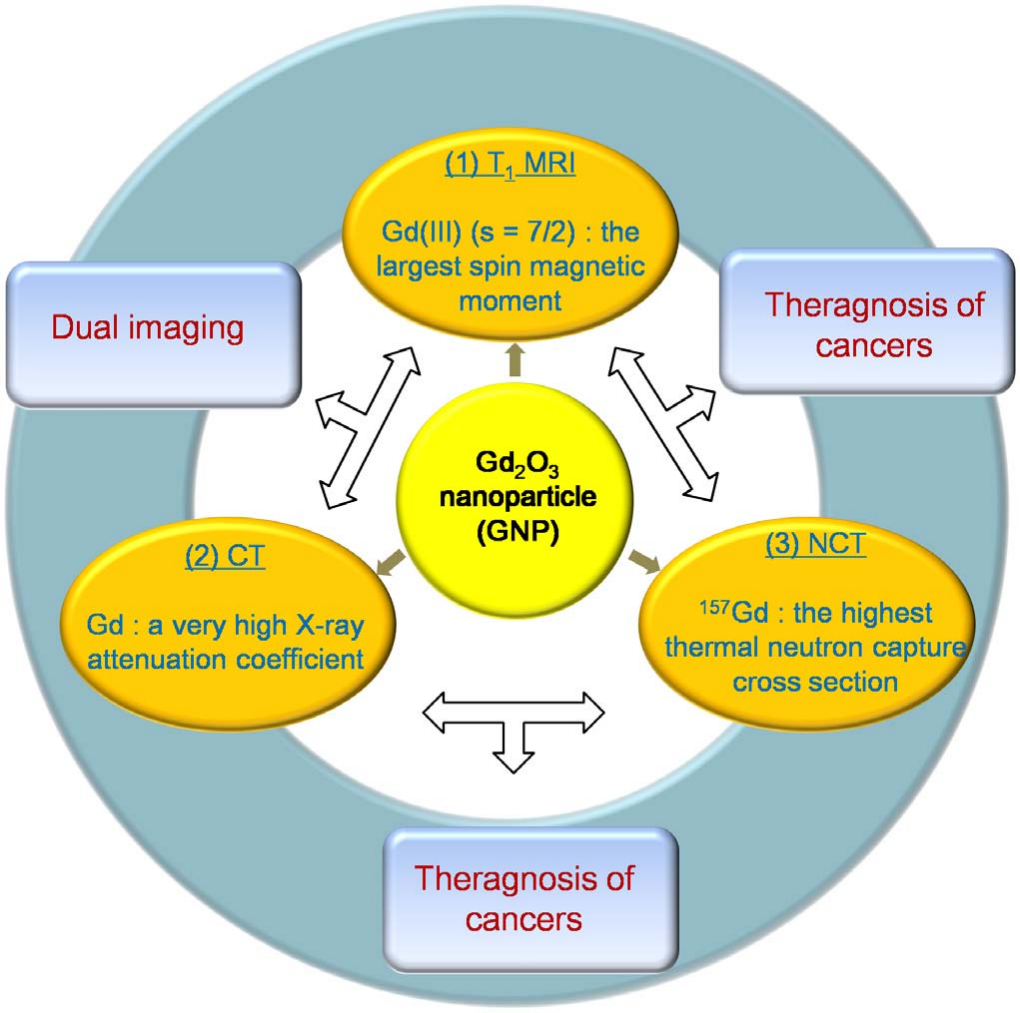
Diagnostic and therapeutic applications of GNP. The three main biomedical applications of GNP are T_1_ MRI, CT, and NCT using the magnetic, X-ray absorption, and thermal neutron capturing properties of Gd, respectively. Combined applications include dual imaging, which is considered in this study, and theragnosis of cancers.

**Figure 2:**
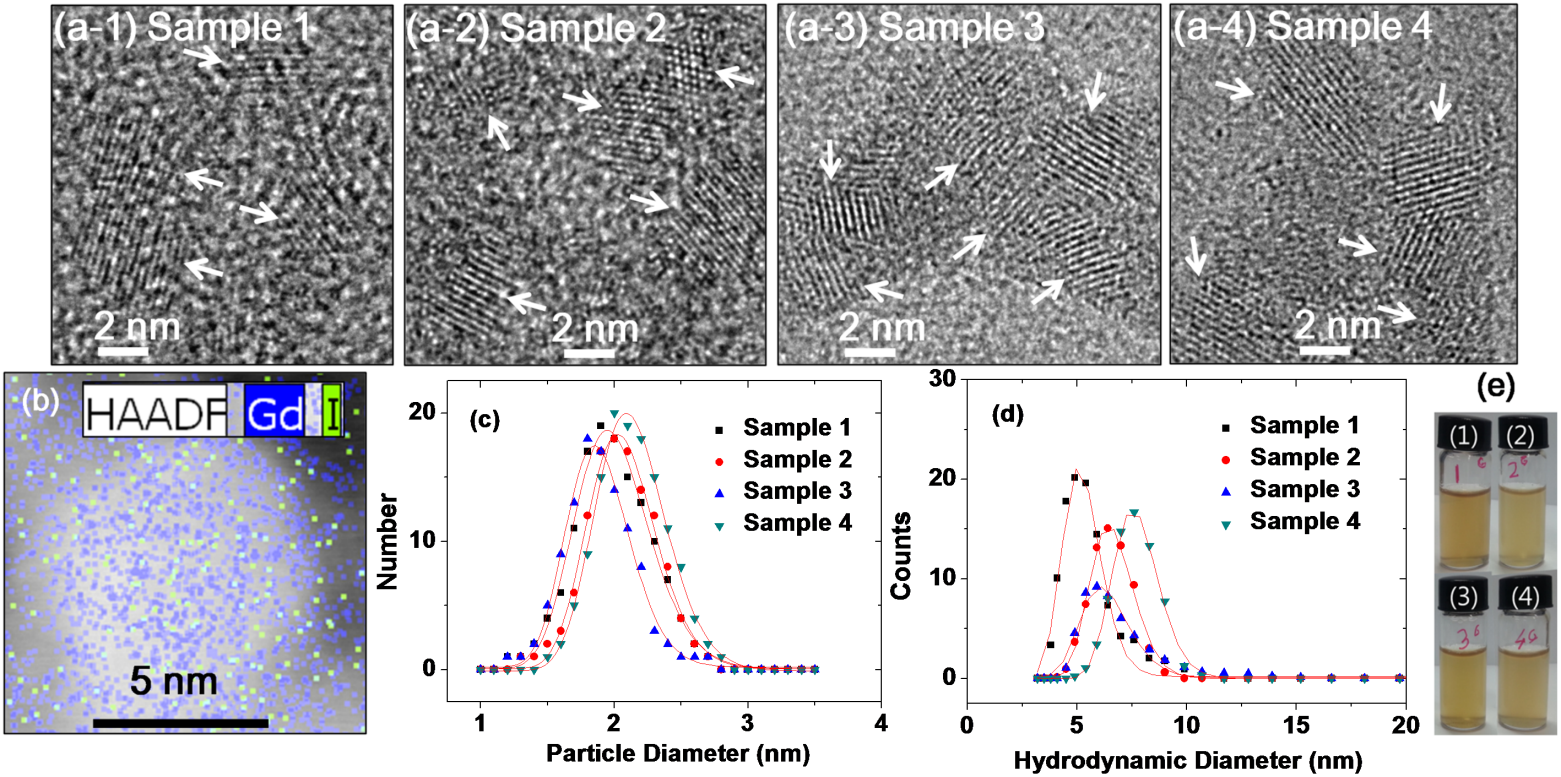
TEM images and DLS patterns. (a-1) to (a-4) HRTEM images of four samples (arrows indicate individual GNPs), (b) a HAADF-STEM image with Gd and I elemental mappings superimposed (Sample 2 was used), (c) particle diameter distributions with log-normal function fits, (d) DLS patterns with log-normal function fits, and (e) photographs of sample solutions with 1 mM Gd in physiological conditions (pH = 7.0, 1 mM glucose, 1 atm, and room temperature) [(1) Sample 1, (2) Sample 2, (3) Sample 3, and (4) Sample 4].

**Figure 3:**
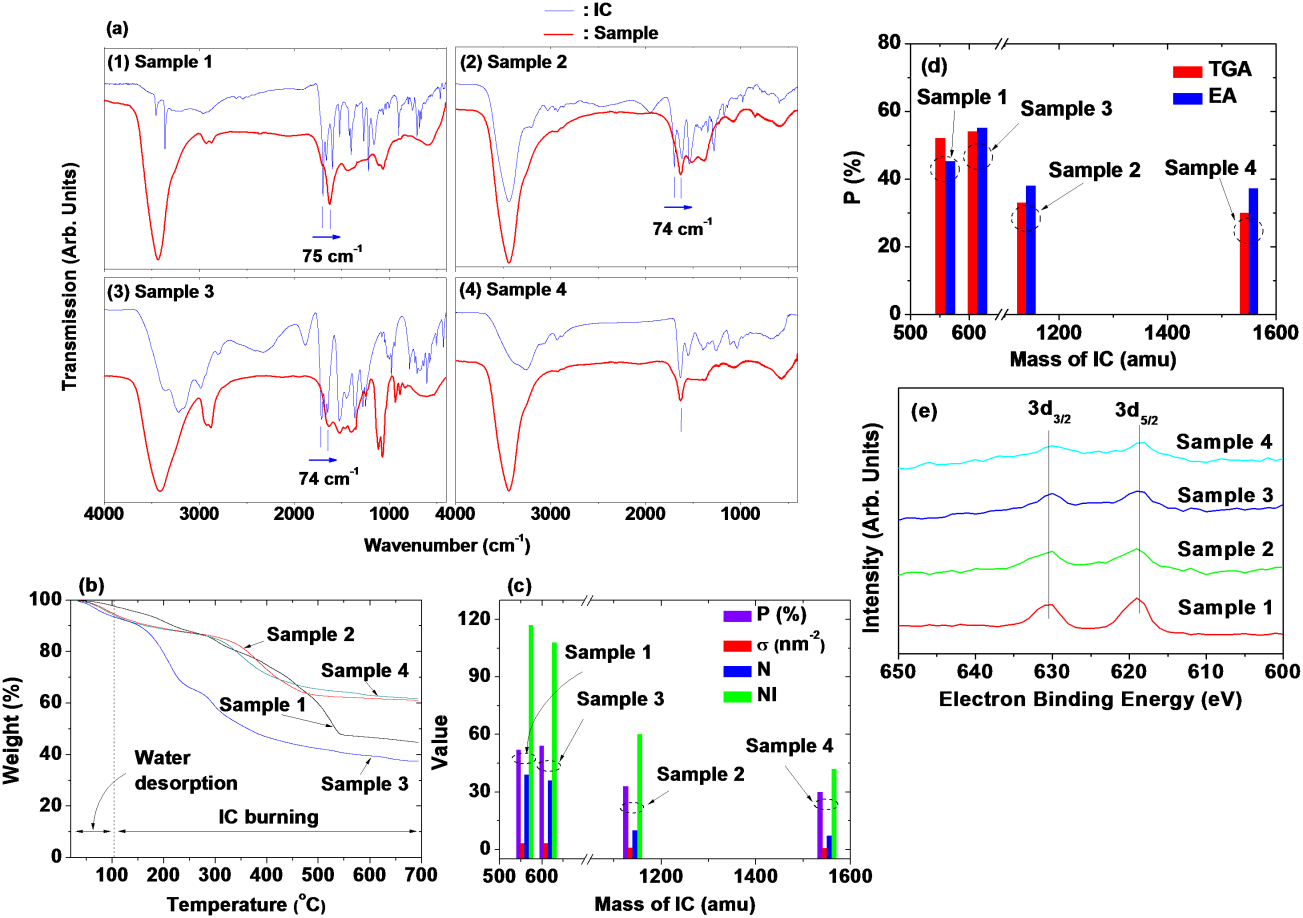
Surface coating results. (a) FT-IR absorption spectra of four powder samples and respective free ICs used for surface coating [arrows in (1) to (3) indicate the red-shifts of bonded COOH with respect to free COOH], (b) TGA curves, (c) surface coating properties (P, σ, N, and NI) as a function of the IC mass (P = weight percent of ICs, σ = grafting density of ICs, N = number of ICs per GNP, and NI = number of iodines per GNP) (here, y-axis is commonly labeled as value for the above surface coating properties), (d) comparison of P values estimated from TGA and EA, and (e) XPS spectra in iodine region (full range XPS spectra are given in Supplementary Information).

Gd is the only element that possesses such diverse and remarkable properties, which are useful for theragnosis for cancers. Therefore, GNPs with the large amount of Gd per GNP will be a powerful theragnostic agent for cancers (Fig. 1)^27^,^28^,^61^. The only shortcoming of Gd is its toxicity^66^. Therefore, GNPs should be coated with water-soluble and biocompatible ligands. Note that this theragnosis might be difficult using the conventional molecular Gd-chelates, because of their low Gd concentrations that could be delivered to cancers^60^.

This study reports the dual imaging capability of GNPs in T_1_ MRI and CT. To this end, four IC-GNP samples were synthesized. Four commercial iodine contrast agents were used as surface coating ligands on GNPs (d_avg_ = ~2.0 nm) to enhance the CT functionalities of the nanoparticles using iodines in the ICs, as well as to make them water-soluble and biocompatible. To evaluate the dual imaging capability of GNPs, the r_1_ values and X-ray absorption of all the samples were measured, and in vivo T_1_ MR and CT images of mice were finally obtained.

Overall, GNPs with a smaller (i.e., less massive) IC coating showed larger r_1_ values and higher X-ray absorption (Fig. 10). This is because more water molecules can access the core GNP for a smaller IC coating, providing the larger r_1_ values^81^, and because GNPs with a smaller IC coating had larger NI values (Table 1), giving higher X-ray absorption. However, all the samples showed larger r_1_ values than commercial T_1_ MRI contrast agents, and higher X-ray absorption than commercial iodine contrast agents. Therefore, all the samples should be potential dual imaging agents in T_1_ MRI and CT.

The dual imaging capability of GNPs was finally confirmed by in vivo T_1_ MR and CT images. That is, positive (or brighter) contrast enhancements in both T_1_ MR and CT images were observed in mice after intravenous injection (Fig. 8). Finally, in vivo comparisons between one of IC-GNP samples and commercial MRI and CT contrast agents were made. More enhanced contrasts in both MR and CT images at the same injection doses were observed, showing the superiority of IC-GNPs to the commercial contrast agents (Fig. 9).

In summary, owing to unique magnetic and X-ray absorption properties of Gd, and a dense population of Gd per GNP, GNPs showed an outstanding dual imaging capability in T_1_ MRI and CT without additional functionalization, which is better than the respective commercial contrast agents. This, when combined with the NCT property of GNPs, will make GNPs a potential theragnostic agent for cancers, which will be investigated in the future.

## Methods

### Chemicals

All the chemicals such as GdCl_3_·*x*H_2_O (>99.9%), NaOH (>99.9%), triethylene glycol (>99%), 5-amino-2,4,6-triiodoisophthalic acid (>95%), iodipamide (>99%), diatrizoic acid (>99%), iodixanol [60% (w/v) in water], and dimethyl sulfoxide (DMSO) (>99.5%) were purchased from Sigma-Aldrich and used as-received. Triply distilled water was used for both washing the nanoparticles and preparing the aqueous sample solutions.

### One-pot synthesis of IC-GNP samples

**Figure 4:**
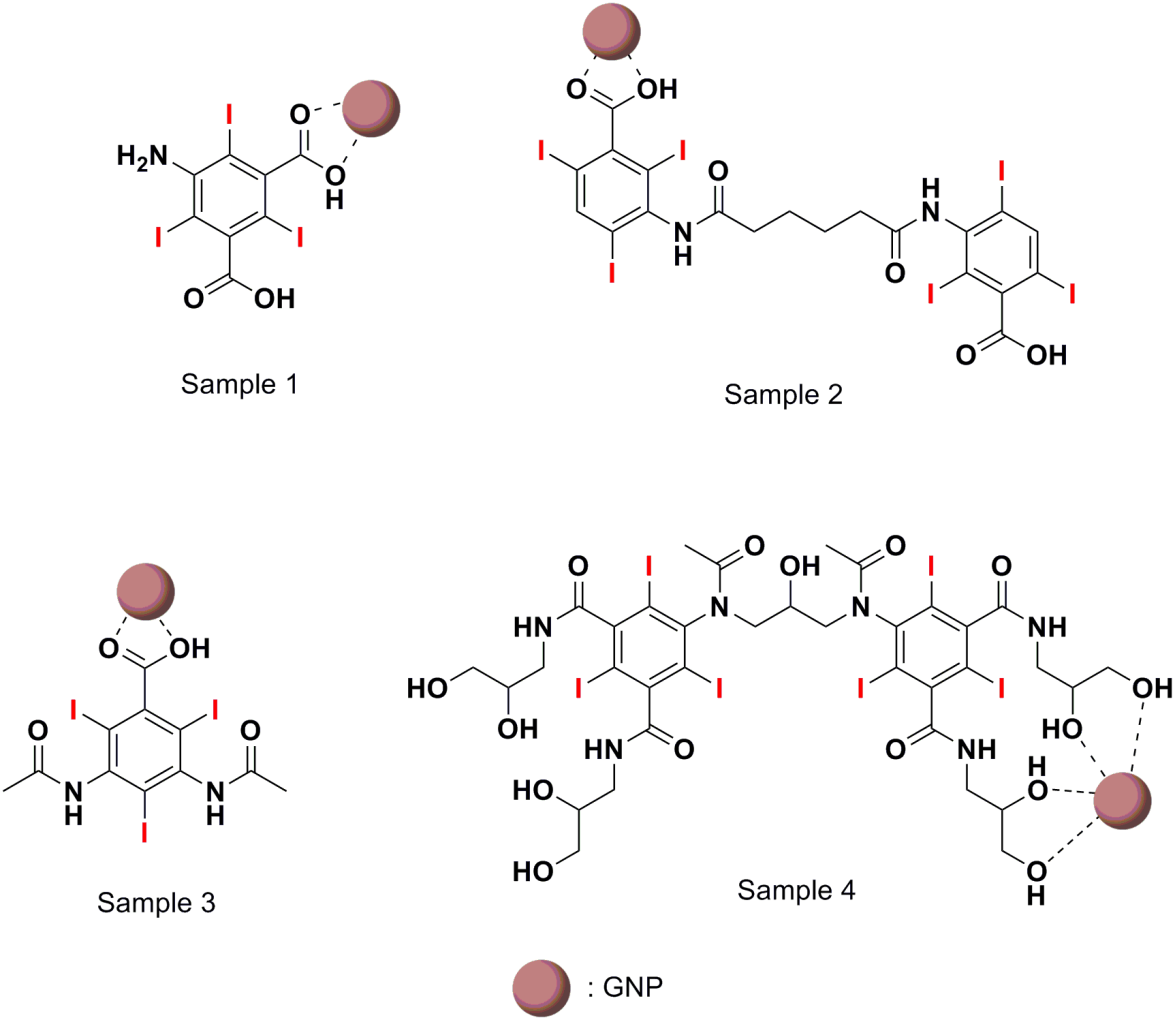
Proposed bonding structures. The most probable bonding structures between ICs and GNPs according to FT-IR absorption spectral results. GNP is not drawn to scale.

**Figure 5:**
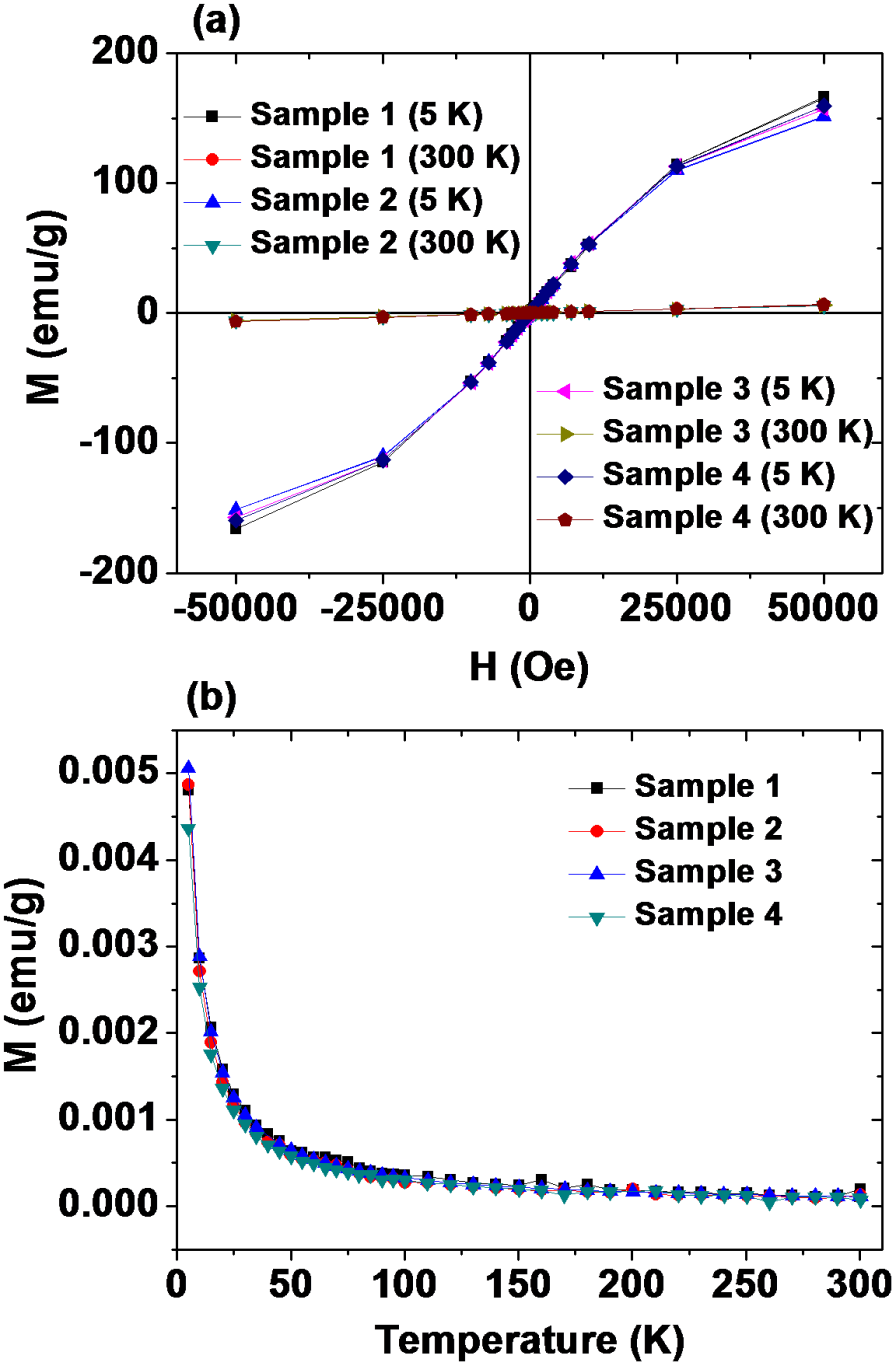
Magnetic properties. Mass corrected (a) M - H curves at T = 5 and 300 K (−5 ≤ H ≤ 5 tesla) and (b) ZFC M - T curves at H = 0 Oe (5 ≤ T ≤ 300 K) of four powder samples.

**Figure 6:**
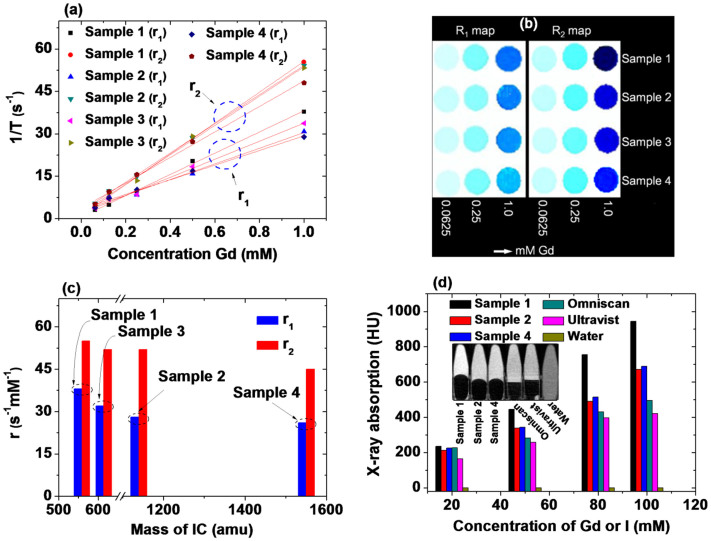
Relaxometric and X-ray absorption results. (a) Plots of 1/T_1_ and 1/T_2_ and (b) R_1_ and R_2_ map images as a function of the Gd concentration [the slopes in plot (a) correspond to the r_1_ and r_2_ values, respectively], (c) plots of r_1_ and r_2_ values as a function of the IC mass, and (d) plots of X-ray absorption as a function of the Gd (or I) concentration [Insets show X-ray phantom images obtained at 100 mM Gd (or I)].

**Figure 7:**
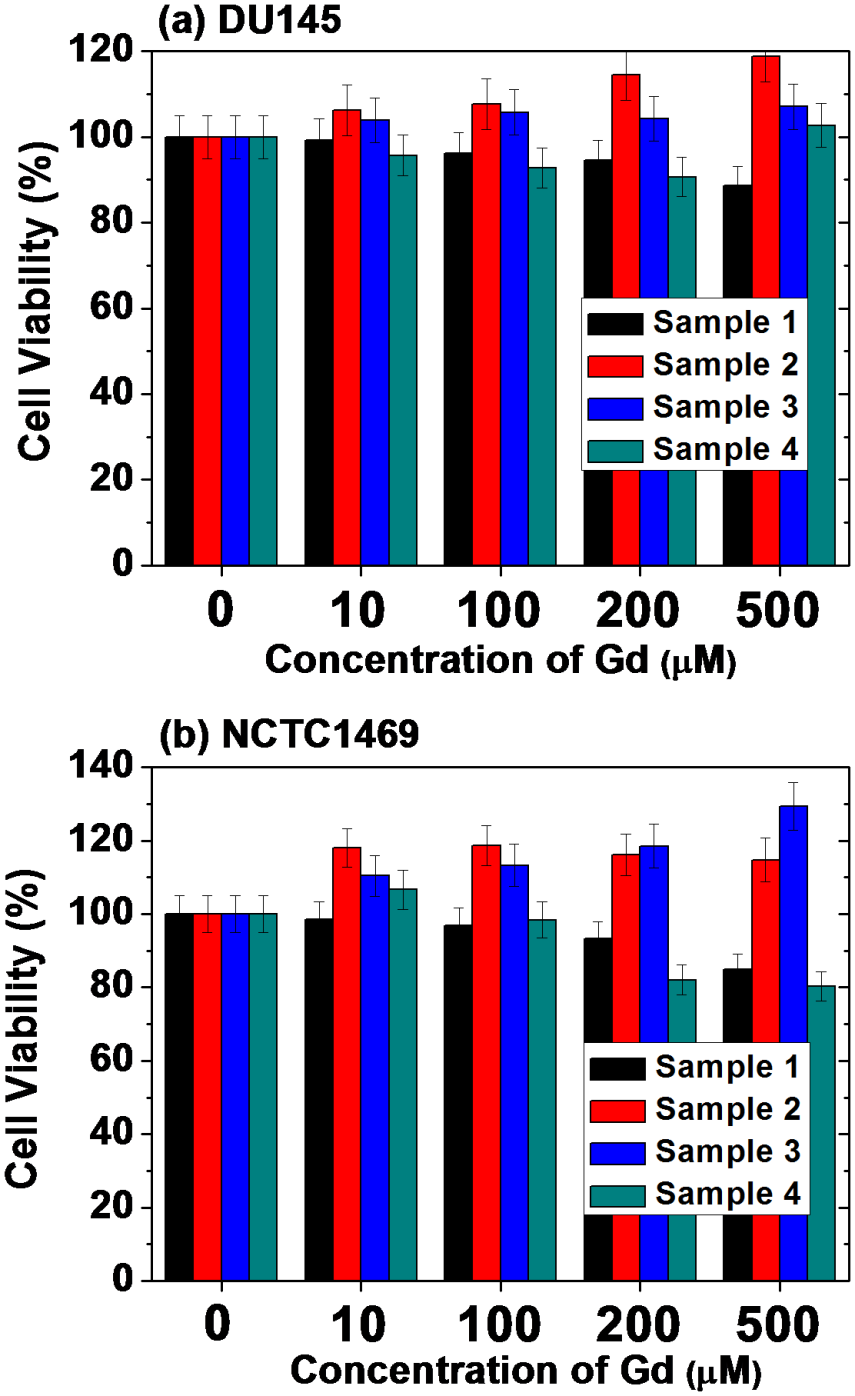
In vitro cytotoxicity results. Normalized cell viabilities of four sample solutions in (a) DU145 and (b) NCTC1469 cell lines are plotted as a function of the Gd concentration, showing no toxicity up to 500 μM Gd.

**Figure 8:**
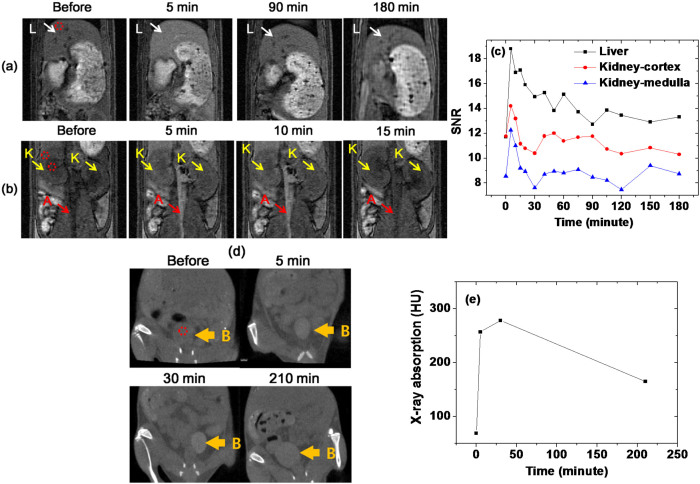
In vivo images of a mouse. (a) T_1_ MR images of a mouse liver (labeled L), (b) kidneys (labeled K) and aorta (labeled A) at 1.5 tesla MR field before and after intravenous injection of Sample 1 into a mouse tail, (c) plots of SNR of ROI in the liver and the cortex and medulla of kidney [positions are labeled with small dotted circles in (a) and (b)] as a function of the time after intravenous injection, (d) in vivo CT images of a mouse bladder (labeled B) before and after intravenous injection of Sample 1 into a mouse tail at an X-ray source voltage of 70 kV, and (e) a plot of X-ray absorption value of ROI in the bladder [position is labeled with a small dotted circle in (d)] as a function of the time after intravenous injection.

**Figure 9:**
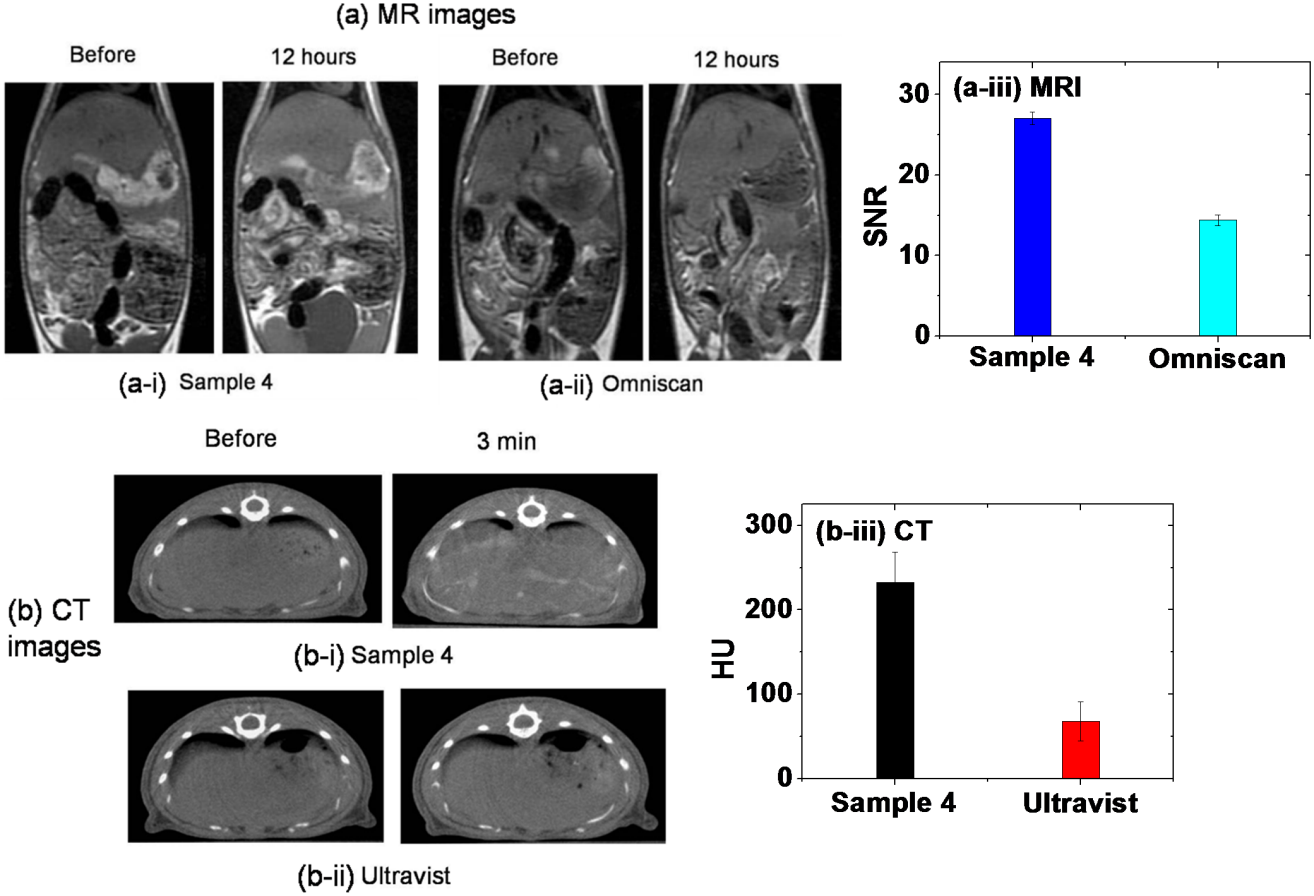
In vivo comparison results between Sample 4 and commercial contrast agents. (a) MR images [(i) the Sample 4, (ii) Omniscan, and (iii) the mean SNR in the liver 12 hours after injection] and (b) CT images [(i) the Sample 4, (ii) Ultravist, and (iii) HU in the liver 3 minutes after injection]. Both experiments showed better contrast enhancements of Sample 4 than the commercial contrast agents at the same injection doses.

**Figure 10:**
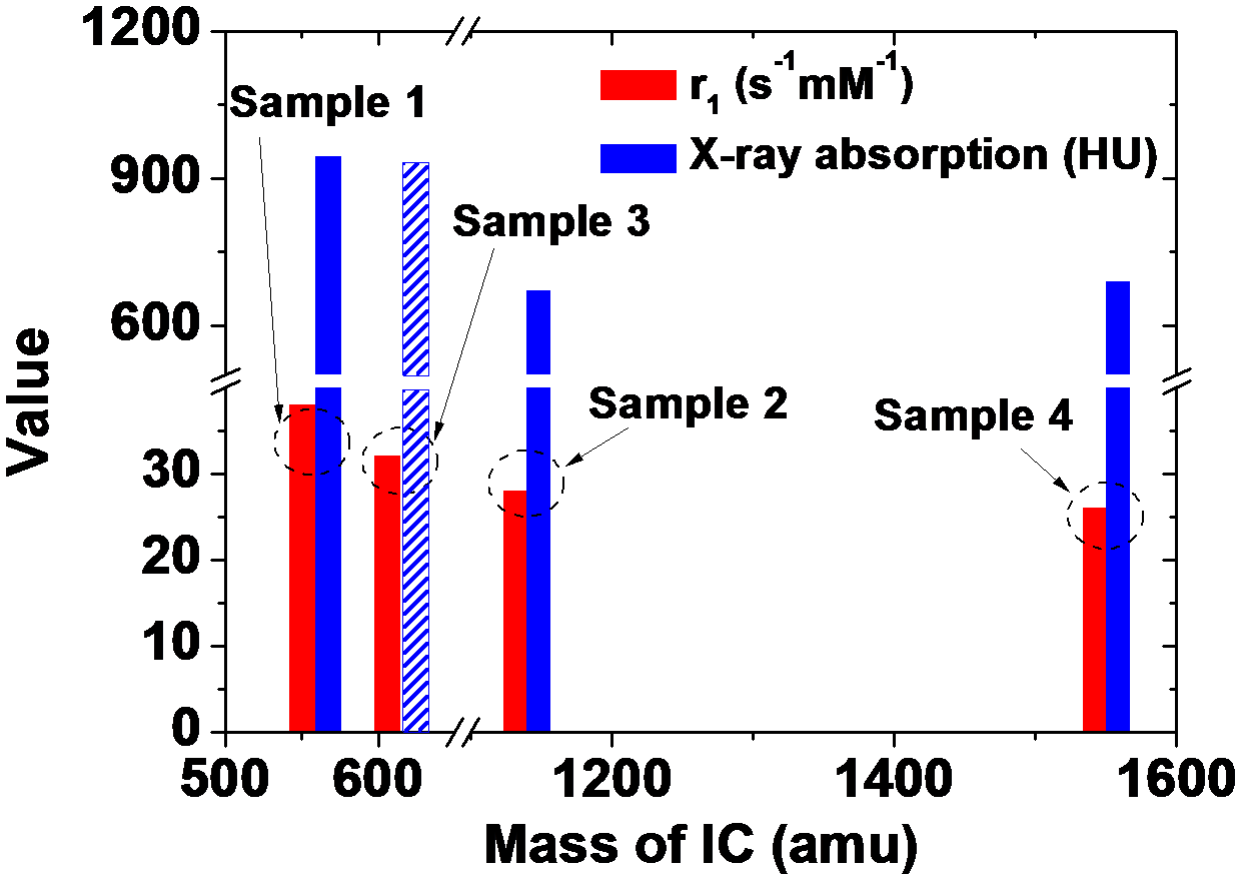
Comparison of T_1_ MRI and CT functionalities between samples. Comparison of r_1_ values and X-ray absorption of all the samples (y-axis is commonly labeled as value for r_1_ value and X-ray absorption). The X-ray absorption of Sample 3 (slashed column) is drawn according to the expectation that it will be comparable to that of Sample 1 because the core is the same GNP and because NI (Sample 1) ≈ NI (Sample 3) as mentioned in the text.

Four IC-GNP samples were prepared by coating four different types of ICs on GNPs. The ICs used were 5-amino-2,4,6-triiodoisophthalic acid, iodipamide, diatrizoic acid, and iodixanol, which are all commercial CT contrast agents (Table 1 and Fig. 4).

**Figure 11:**
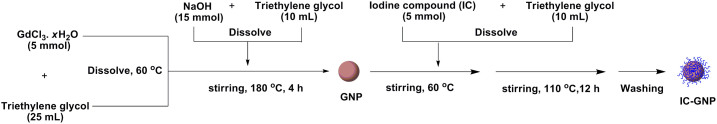
One-pot synthesis. One-pot synthesis procedure for four IC-GNP samples.

The IC-GNP samples were synthesized in one-pot using the procedure shown in Fig. 11. Three separate solutions were prepared: (i) a precursor solution made of 5 mmol of GdCl_3_·*x*H_2_O in 25 mL of triethylene glycol, (ii) a NaOH solution made of 15 mmol of NaOH in 10 mL of triethylene glycol, and (iii) an IC solution made of 5 mmol of IC in 10 mL of triethylene glycol (in the case of iodipamide, five drops of DMSO were also added to the solution to completely dissolve the iodipamide). The precursor solution was heated to 60°C with magnetic stirring under atmospheric conditions until the precursor was completely dissolved in the solvent. The NaOH solution was then added to the precursor solution. The mixed solution was magnetically stirred at 180°C for 4 hours. For surface coating, the solution temperature was then lowered to 60°C and an IC solution was added slowly to the above solution. The temperature of the solution was again raised to 110°C, and the solution was magnetically stirred for an additional 12 hours. To wash the IC-GNP samples with triply distilled water, the solution was then cooled to room temperature and transferred to a 1 L beaker containing 500 mL of triply distilled water. It was then magnetically stirred for 10 minutes and stored for a week to let the IC-GNP samples settle to the bottom of the beaker. The clear supernatant was decanted and the remaining sample was again diluted with 500 mL of triply distilled water. This washing process was repeated three times. A half volume of each sample was dried in air to obtain powder samples for various characterizations, and the remaining half volume was diluted with triply distilled water to obtain solution samples.

### Measurements of particle diameter, hydrodynamic diameter, and crystal structure

The particle diameters of the IC-GNP samples were measured using an HRTEM (FEI, Titan G2 ChemiSTEM CS Probe) operating at an acceleration voltage of 200 kV. For the measurements, one drop of each sample dispersed in ethanol was dropped onto a carbon film supported by a 200 mesh copper grid (PELCO No.160, TED PELLA, INC.) placed on a filter paper using a micropipette (Eppendorf, 2–20 μL). The copper grid with the sample was left in air to dry for an hour at room temperature. The copper grid with the sample was then mounted inside the HRTEM for measurement.

The Gd concentration of each sample solution was determined using an inductively coupled plasma atomic emission spectrometer (Thermo Jarrell Ash Co., IRIS/AP). All the samples were pre-treated with acids to completely dissolve the nanoparticles in solution before measurement.

The hydrodynamic diameters of the IC-GNP samples dispersed in triply distilled water were measured using a DLS particle size analyzer (UPA-150, Microtrac). The sample solution concentration was ~ 0.05 mM Gd.

The crystal structure of the IC-GNP powder samples before and after TGA analysis was measured using a powder XRD spectrometer (Philips, X-PERT PRO MRD) with unfiltered CuKα (λ = 1.54184 Å) radiation. The scanning step and scan range in 2θ were 0.033° and 15–100°, respectively.

### Surface coating analysis

The surface coating of GNPs with ICs was investigated using an FT-IR absorption spectrometer (Mattson Instruments, Inc., Galaxy 7020A). For the measurements, powder samples were dried on a hot plate at ~40°C for a week to remove moisture from them. Pellets of dried powder samples in KBr were prepared, and FT-IR absorption spectra were recorded between 400 and 4000 cm^−1^.

The amount of the IC coated on the GNP surface was estimated using a TGA instrument (TA Instruments, SDT-Q 600). Because organic compounds burn out below 400°C, TGA curves for each powder sample were scanned between room temperature and 700°C under air flow. The amount of surface coating for each sample was estimated from the mass drop in its TGA curve after an initial mass drop between room temperature and ~105°C due to water desorption was subtracted.

The elemental analyses of surface coated ICs on GNP surfaces were carried out using both the EA (ThermoFisher, Flash 2000) and XPS (ULVAC-PHI, Quantera SXM). Powder samples were used for both measurements. For XPS measurements, powder samples were loaded onto carbon tapes and the spectra were scanned between 0 and 1200 eV in electron binding energy (EBE) with the accumulation time of 30 to 50 minutes. The EA was used to measure the C, H, O, and N in weight percents, whereas the XPS was used to measure the C, O, N, I, and Gd in atomic percents.

### Magnetic property measurement

The magnetic properties of all the powder samples were measured using a superconducting quantum interference device magnetometer (Quantum Design, MPMS-7). Both the M - H curves (−5 ≤ H ≤ 5 tesla) at T = 5 and 300 K and the ZFC M - T curves (5 ≤ T ≤ 300 K) at H = 0 Oe were recorded. To measure both curves, each weighed powder sample was loaded into a nonmagnetic gelatin capsule. The very small diamagnetic contribution of the capsule had a negligible effect on the overall M, which was dominated by the sample. The net M value for the GNPs in each powder sample was obtained by multiplying the measured M value by the weight percent of GNPs in the sample estimated from the corresponding TGA curve.

### Relaxometric measurement

Both the T_1_ and T_2_ relaxation times and R_1_ and R_2_ map images were measured using a 1.5 tesla MRI scanner (GE 1.5 T Signa Advantage, GE Medical Systems) equipped with a knee coil (EXTREM). Five aqueous dilute solutions (1, 0.5, 0.25, 0.125, and 0.0625 mM Gd) were prepared per sample by diluting each concentrated sample solution with triply distilled water. These dilute solutions were then used to measure both the T_1_ and T_2_ relaxation times and R_1_ and R_2_ map images. The r_1_ and r_2_ water proton relaxivities of each sample were then estimated from the slopes of plots of 1/T_1_ and 1/T_2_, respectively, versus the Gd concentration. T_1_ relaxation time measurements were carried out using an inversion recovery method. In this method, the inversion time (TI) was varied at 1.5 tesla and the MR images were acquired at 35 different TI values ranging from 50 to 1750 ms. The T_1_ relaxation times were then obtained from the nonlinear least-square fits to the measured signal intensities at various TI values. For T_2_ relaxation time measurements, the Carr−Purcell−Meiboon−Gill pulse sequence was used for multiple spin−echo measurements. Then, 34 images were acquired at 34 different echo time (TE) values ranging from 10 to 1900 ms. The T_2_ relaxation times were obtained from the nonlinear least-square fits to the mean pixel values for the multiple spin−echo measurements at various TE values.

### Cell viability measurement

The cytotoxicity of each sample was measured using a CellTiter-Glo Luminescent Cell Viability Assay (Promega, WI, USA). In this assay, the intracellular adenosine triphosphate was quantified using a luminometer (Victor 3, Perkin Elmer). DU145 and NCTC1469 cell lines were used as test cells. Each cell line was seeded onto a separate 24-well cell culture plate and incubated for 24 hours (5 × 10^4^ cell density, 500 mL cells per well, 5% CO_2_, and 37°C). Four dilute solutions were prepared per sample by diluting each concentrated sample solution with a sterile phosphate buffer saline solution. Each test cell was treated by ~2 μL of one of the dilute solutions. The final Gd concentrations in the four treated cells were 10, 100, 200, and 500 μM per sample. The treated cells were then incubated for 48 hours. The cell viabilities of treated cells were measured twice to obtain the average values and then normalized with respect to a control cell that was not treated with a sample solution.

### Animal experiment

All the animal experiments using mice were approved by the animal research committee of Kyungpook National University and conducted in accordance with its rules.

### In vivo T_1_ MR image measurement

In vivo T_1_ MR images of mice were acquired using the same MRI scanner used for the relaxometric measurements. ICR female mice with a weight of ~30 g were used for the T_1_ MR image measurements. For imaging, a mouse was anesthetized using 1.5% isoflurane in oxygen. Measurements were made before and after injection of a sample solution into a mouse tail vein. The injection dose was typically ~0.1 mmol Gd/kg. After measurement, the mouse was revived from anaesthesia and placed in a cage with free access to food and water. During the measurement, the temperature of the mouse was maintained at ~37°C using a warm water blanket. The parameters used for the measurements are as follows: the H = 1.5 tesla, the T = 37°C, the NEX = 8, the FOV = 100 mm, the phase FOV = 0.5, the matrix size = 256 × 160, the slice thickness = 1 mm, the spacing gap = 0.5 mm, the pixel bandwidth = 15.63 Hz, the TR = 14.4 ms, and the TE = 4.3 ms.

### Phantom image and X-ray absorption measurements

X-ray phantom images were acquired using a micro-CT scanner (Siemens, Inveon). Four dilute solutions (20, 50, 80, and 100 mM Gd) were prepared per sample by diluting each concentrated sample solution with triply distilled water. A phantom image of water served as a reference with 0.0 HU, and those of the commercial contrast agents Ultravist (20, 50, 80, and 100 mM I) and Omniscan (20, 50, 80, and 100 mM Gd) were also measured for comparison. The X-ray absorption of each dilute solution was estimated in HU with respect to water. The parameters used for the measurements are as follows: the X-ray source current = 400 μA, the X-ray source voltage = 70 kV, the imaging time per frame = 200 ms, and the reconstructed image size = 512 × 512.

### In vivo CT image measurement

In vivo CT images of mice were acquired using the same micro-CT scanner used for the phantom image measurements. ICR female mice with a weight of ~30 g were used for the measurements. The injection dose was typically ~0.53 mmol Gd/kg. For imaging, a mouse was anesthetized using 1.5% isoflurane in oxygen. Measurements were made before and after injection of a sample solution into a mouse tail vein. After measurement, the mouse was revived from anaesthesia and placed in a cage with free access to food and water. The parameters used for measurements are as follows: the X-ray source current = 400 μA, the X-ray source voltage = 70 kV, the imaging time per frame = 200 ms, and the reconstructed image size = 512 × 512.

## Author Contributions

M.W.A. synthesized and characterized the samples. W.X. and S.J.K. characterized the samples. J.S.B. and Y.C. measured the relaxivities, and MR and CT images. J.E.B. and K.S.C. measured the cellular toxicities. J.A.P. obtained the X-ray phantom and CT images. T.J.K. and G.H.L. led the project and G.H.L. wrote the paper.

## Supplementary Material

Supplementary Information Supplementary Information

## Figures and Tables

**Table 1:** Average particle diameter (d_avg_), average hydrodynamic diameter (a_avg_), surface coating results (P, σ, N, NI), and magnetization (M)

								M at H = 5 tesla (emu/g)	
Sample	Surface coating IC (Molecular formula, molecular mass)	d_avg_ (nm)	a_avg_ (nm)	P (%)	σ (nm^−2^)	N	NI	5 K	300 K
Sample 1	5-Amino-2,4,6-triiodoisophthalic acid^a^ (C_8_H_4_I_3_NO_4_, 558.84 amu)	2.0	5.2	52	3.07	39	117	165.4	6.6
Sample 2	Iodipamide^b^ (C_20_H_14_I_6_N_2_O_6_, 1139.76 amu)	2.1	6.6	33	0.74	10	60	151.1	5.8
Sample 3	Diatrizoic acid^c^ (C_11_H_9_I_3_N_2_O_4_, 613.91 amu)	1.9	6.3	54	3.19	36	108	157.1	6.1
Sample 4	Iodixanol^d^ (C_35_H_44_I_6_N_6_O_15_, 1550.18 amu)	2.1	7.7	30	0.51	7	42	159.2	6.2

^a^A basic molecule used for the synthesis of commercial iodine contrast agents such as Iohexol (trade name: Omnipaque, GE Heanthcare Inc.) and Iopamidol (trade name: Isovue, Bracco, USA).

^b^Also called Adipiodone (brand name: Sinografin, Bracco, USA).

^c^Brand name: Hypaque, GE Healthcare Inc., USA.

^d^Trade name: Visipaque, GE Healthcare Inc., USA.

**Table 2:** r_1_ and r_2_ values of various chemicals

Chemical	H (tesla)	T (°C)	r_1_ (s^−1^mM^−1^)	r_2_ (s^−1^mM^−1^)	r_2_/r_1_	Ref.
Sample 1	1.5	22	38	55	1.4	This work
Sample 2	1.5	22	28	52	1.9	This work
Sample 3	1.5	22	32	52	1.6	This work
Sample 4	1.5	22	26	45	1.7	This work
Gd-DTPA	0.47	25	3.8	4.2	1.1	30
Gd-DOTA	0.47	25	4.2	4.6	1.1	30
