# Supplementary material for: Potential dual imaging nanoparticle: Gd2O3 nanoparticle
Source: Sci Rep. 2015 Feb 24;5:8549. doi: 10.1038/srep08549 (PMC4338476; doi:10.1038/srep08549)
Supplement: Supplementary Information [file srep08549-s1.doc]

**Supplementary Information**

**Title**: Potential dual imaging nanoparticle: Gd_2_O_3_ nanoparticle

**Authors**: Md. Wasi Ahmad, Wenlong Xu, Sung June Kim, Jong Su Baeck, Yongmin Chang, Ji Eun Bae, Kwon Seok Chae, Ji Ae Park, Tae Jeong Kim & Gang Ho Lee

**(1) XRD patterns before and after TGA analysis**

The XRD patterns of the as-prepared powder samples and after TGA analysis are shown in Fig. S1. Very broad XRD patterns of the as-prepared powder samples are likely because of their ultrasmall particle diameters^1^. After TGA analysis up to ~ 700 ^o^C, however, sharp peaks corresponding to (211), (222), (400), (440), and (622) of cubic Gd_2_O_3_, appeared as shown at the top in the XRD patterns. The estimated cell constant (a) of the TGA-analyzed powder sample is 10.81 Å, which is consistent with the reported value (= 10.813 Å) of cubic Gd_2_O_3_ (card no. 43-1014, PCPDFWIN)^2^. The measured peak positions and cell constant are provided in Table S1.

**Figure S1.** XRD patterns of the as-prepared powder samples and after TGA analysis.

| **Table S1. Peak assignment with Miller index (hkl) and cell constant (a).** | |
| --- | --- |
| hkl | 2θ (^o^) |
| 211 | 20.03 |
| 222 | 28.55 |
| 400 | 33.12 |
| 440 | 47.50 |
| 622 | 56.44 |
| a = 10.81 Å | |

**(2) FT-IR absorption spectra and peak assignments**

FT-IR absorption spectra of four powder samples and respective free ICs are provided in Figs. S2a - d. Some important absorption peaks are labeled with “*” and their transitions are assigned in Table S2.

**Figure S2.** FT-IR absorption spectra of four powder samples and respective free ICs.

| **Table S2.** **Assignment of vibrational absorption peaks.** | | | | | |
| --- | --- | --- | --- | --- | --- |
| Sample | IC-GNP | |  | Free IC | |
|  | Peak  (cm^-1^) | Assignment |  | Peak  (cm^-1^) | Assignment |
| Sample 1 | 3432  1630  1440  1069  590 | (OH)_S_  (COOH)_AS_  (COOH)_SS_  (CO)_S_  (GdO)_S_ |  | 3459  3360  1698  1662  1595  1523  1159  686 | (NH_2_)_AS_  (NH_2_)_S_  (COOH)_AS_  (NH_2_)_B_  (C=C)_S_  (COOH)_SS_  (CO)_S_  (CI)_S_ |
| Sample 2 | 3436  1632  1511  1381  1070  584 | (OH)_S_  (COOH)_AS_ / (C=O)_S_  (NH)_B_ / (C=C)_S_  (COOH)_SS_  (CO)_S_  (GdO)_S_ |  | 3436  1697  1619  1539  591 | (OH)_S_  (COOH)_AS_  (NH)_B_ / (C=O)_S_  (COOH)_SS_  (CI)_S_ |
| Sample 3 | 3417  2920  2875  1639  1525  1397  1117  1071  610 | (OH)_S_  (CH)_S_  (CH)_S_  (COOH)_AS_ / (C=O)_S_  (NH)_B_ / (C=C)_S_  (COOH)_SS_  (CO)_S_  (CO)_S_  (GdO)_S_ |  | 3370  3218  2982  1712  1671  1648  1528  1449  1001  680 | (OH)_S_  (NH)_S_  (CH)_S_  (COOH)_AS_  (C=O)_S_  (NH)_B_  (COOH)_SS_  (C=C)_S_  (CO)_S_  (CI)_S_ |
| Sample 4 | 3440  1631  1380  1074  568 | (OH)_S_  (C=O)_S_  (NH)_B_ / (C=C)_S_  (CO)_S_  (GdO)_S_ |  | 3372  3260  1631  1548  1397  1112  1032  668 | (OH)_S_  (NH)_S_  (C=O)_S_  (NH)_B_  (C=C)_S_  (CO)_S_  (CO)_S_  (CI)_S_ |
| S: stretch, SS: symmetric stretch, AS: asymmetric stretch, B: bend | | | | | |

**(3) Elemental analyses**

The surface coated materials were analyzed using both the elemental analyzer (EA) and the X-ray photoelectron spectrometer (XPS). First, the EA results are provided in Table S3. The EA (C, H, O, N) elemental analyses show that the total weight percents are 45.2, 38.0, 55.1, and 37.2 for samples 1, 2, 3, and 4, respectively, which are roughly consistent with the respective TGA data given in Table 1 in the text. However, the C, H, O, N weight percent ratios were not consistent with those calculated from the molecular formula of IC because the surface coated materials also contained some triethylene glycol (i.e., solvent) and moisture in addition to ICs.

| **Table S3. Elemental analysis from EA.** | | | | | |
| --- | --- | --- | --- | --- | --- |
| **Sample number** | **Element**  **(weight percent, %)** | | | | |
|  | **C** | **H** | **O** | **N** | **Total** |
| 1 | 19.3 | 2.8 | 22.8 | 0.3 | 45.2 |
| 2 | 11.4 | 1.9 | 24.6 | 0.1 | 38.0 |
| 3 | 23.2 | 4.0 | 27.8 | 0.1 | 55.1 |
| 4 | 12.1 | 2.0 | 23.0 | 0.1 | 37.2 |

The XPS spectra are provided in Fig. S3. The XPS spectra clearly showed iodines in all samples, confirming the surface coating of GNPs with ICs in all samples. That is, the finger print transitions of iodine at 619 (3d_5/2_) and 630 eV (3d_3/2_) regions were observed in all samples. The other transitions of C, O, N, and Gd were also observed. Here, the transitions of N were weakly observed.

**Figure S3.** XPS spectra of four powder samples and carbon tape (i.e., background).

The XPS spectra of TGA analyzed samples were also recorded to investigate the remaining elements in the nanoparticles after TGA analysis (Fig. S4a and b). Here, the detected C signals come from the carbon tape (i.e., background signal). Signal intensities were generally lower than those in Fig.S3, owing to small amounts of samples after TGA analyses. As can be seen in Fig. S4b, iodines still appeared in XPS spectra of the TGA analyzed samples, likely owing to solid compound formations with either oxygen or gadolinium during TGA analysis. These caused the overestimation of net Gd_2_O_3_ masses in sample masses from TGA curves. The full transition assignments of all as-synthesized and TGA analyzed samples are provided in the electron binding energy (EBE) scale in Table S4. All transition EBEs are consistent with literature values^3^.

**Figure S4.** XPS spectra of four TGA analyzed powder samples: (a) full scan and (b) iodine region.

| **Table S4. Transition assignments in XPS spectra.** | | | |
| --- | --- | --- | --- |
| Transition | Element | EBE (eV) | |
|  |  | Observed | Literature^3^ |
| 1 | Gd | 9.8 (4f) | 8 |
| 2 | Gd | 22.9 (5p) | 21 |
| 3 | I | 50.6 (4d_3/2_ + 4d_5/2_) | 49 (4d_5/2_), 51 (4d_3/2_) |
| 4 | Gd | 143.5 (4d) | 140 |
| 5 | C | 286.0 (1s) | 285 |
| 6 | N | 398.3 (1s) | 400.0 |
| 7 | O | 532.6 (1s) | 531 |
| 8 | I | 618.8 (3d_5/2_) | 619 |
| 9 | I | 630.2 (3d_3/2_) | 630 |
| 10 | O | 978.0 (KLL) | 979 |
| 11 | Gd | 1187.6 (3d_5/2_) | 1186 |

**(4) X-ray phantom images at various Gd (or I) concentrations**

X-ray phantom images of four samples at an X-ray source voltage of 70 kV and at concentrations of 20, 50, 80, 100 mM Gd, are provided in Fig. S5. X-ray phantom images of water, Omniscan (Gd-chelate T_1_ MRI contrast agent), and Ultravist (iodine CT contrast agent) are also provided at the same Gd (or I) concentrations for comparison. Water is a reference with 0.0 HU. As given in Fig. S5, the contrasts of X-ray phantom images become brighter with increasing Gd (or I) concentration because the X-ray absorption increases with increasing Gd (or I) concentration. The contrasts of samples are brighter than those of Omniscan and Ultravist because the samples have both Gd and I, whereas Omniscan has only Gd and Ultravist has only I, and because Gd more strongly absorbs X-ray radiation than I.


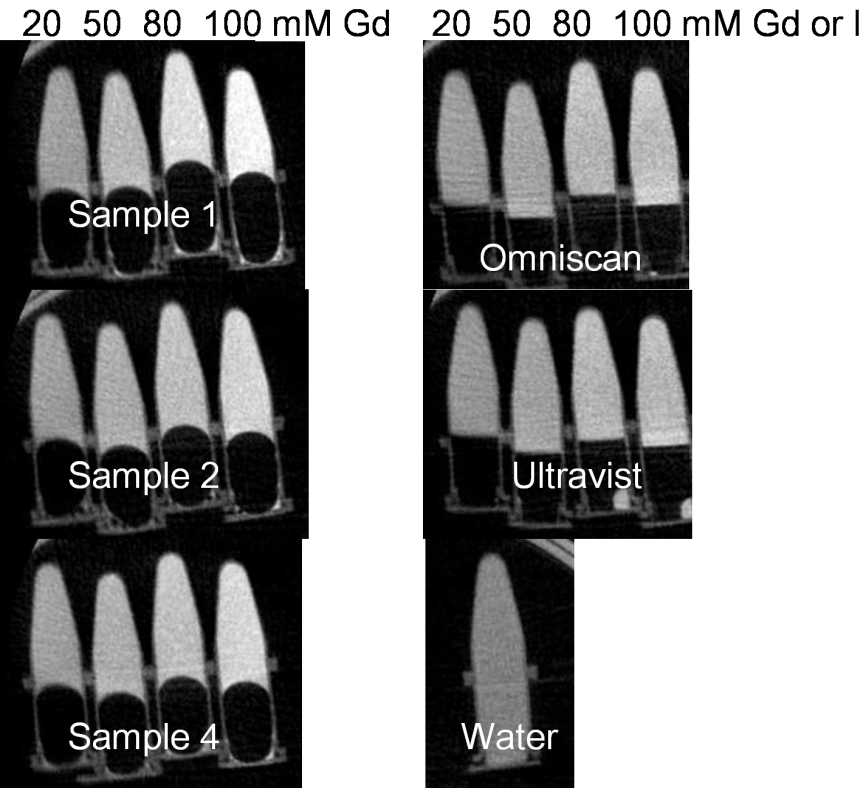


**Figure S5.** X-ray phantom images at an X-ray source voltage of 70 kV and at concentrations of 20, 50, 80, 100 mM Gd (or I).

**(5) In vivo T_1_ MR images of an ICR mouse at 1.5 tesla MR field**

Additional T_1_ MR images at 1.5 tesla MR field are provided in Figs. S6a and b. These images were acquired after intravenous injection of Sample 1 into a mouse tail. Approximately 0.1 mmol Gd/kg was injected into a mouse tail vein. Appreciably positive (or brighter) contrast enhancements were observed in the mouse liver after injection, but returned almost to the initial contrast (i.e., the contrast before injection), 90 minutes after injection (Fig. S6a). Appreciably positive contrast enhancements in the mouse aorta (labeled A) and slightly positive contrast enhancements in the mouse kidneys (labeled K) were also observed after injection, but all of them returned almost to the respective initial contrasts 15 minutes after injection (Fig. S6b), due likely to the excretion of nanoparticles from the respective organs. These results confirm that the sample solution is a potential T_1_ MRI contrast agent.


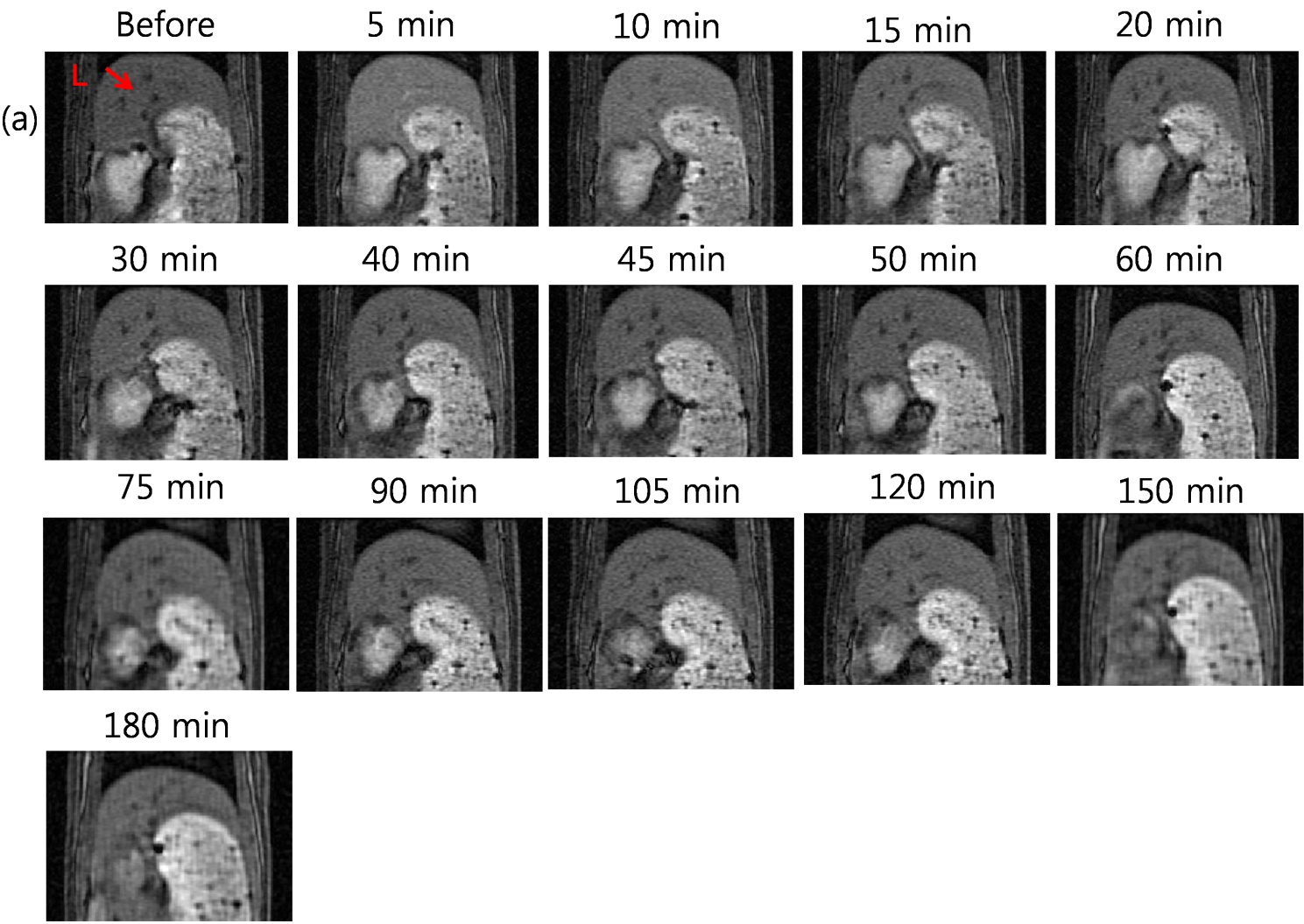


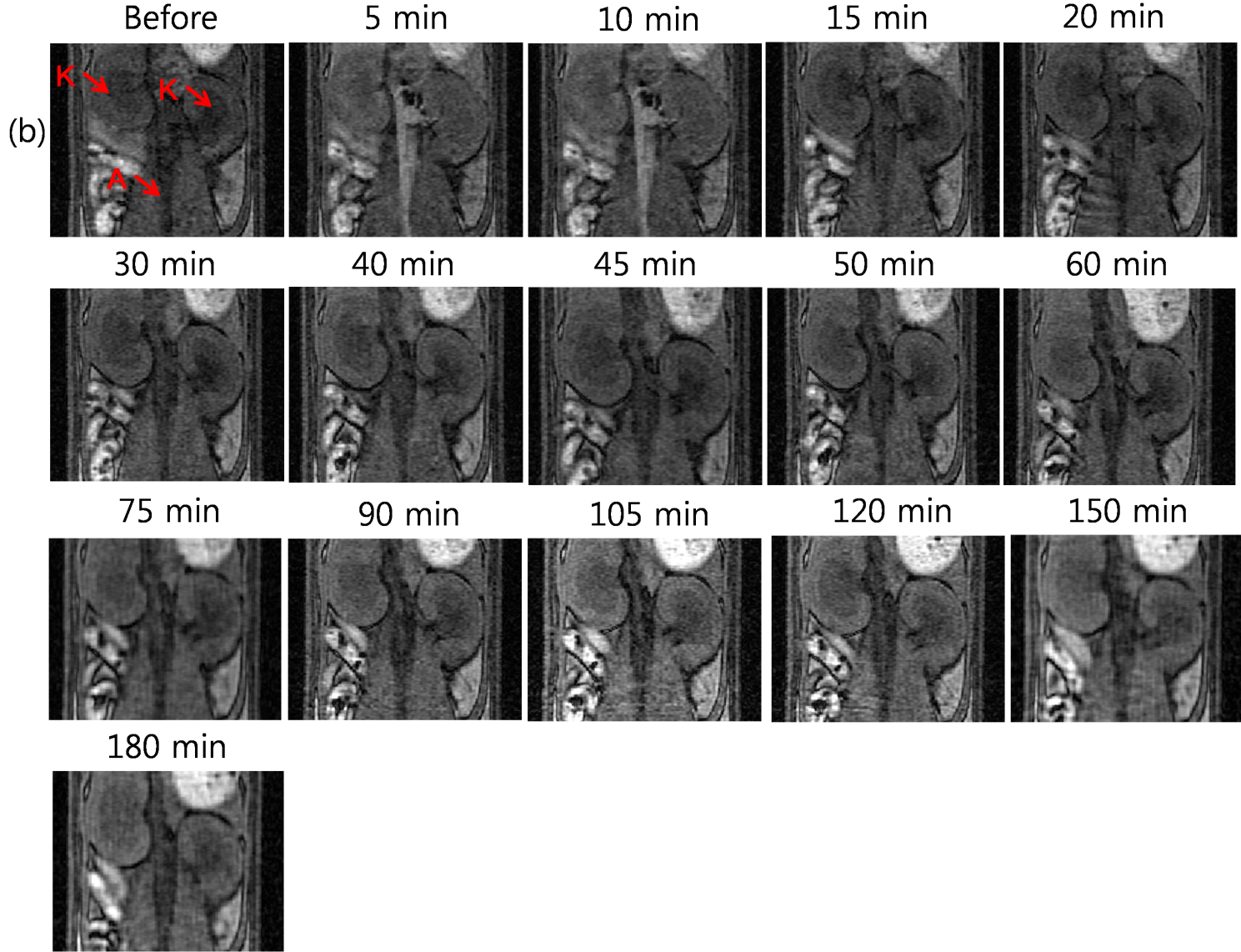


**Figure S6.** (a) In vivo T_1_ MR images of the mouse liver (labeled L) and (b) kidneys (labeled K) and aorta (labeled A) before and after intravenous injection.

**(6) In vivo CT images of an ICR mouse at an X-ray source voltage of 70 kV**

Additional in vivo CT images at an X-ray source voltage of 70 kV are provided in Fig. S7. These images were acquired after intravenous injection of Sample 2 into a mouse tail. Approximately 0.53 mmol Gd/kg was injected into a mouse tail vein, and in vivo CT images were acquired before and after injection. Brighter contrast enhancements were observed in the mouse bladder (labeled B) after injection, and maintained up to more than 210 minutes after injection. These contrast enhancements in the bladder show that the sample solution is excreted through bladder as urine, which is neceaasry for clinical applications.


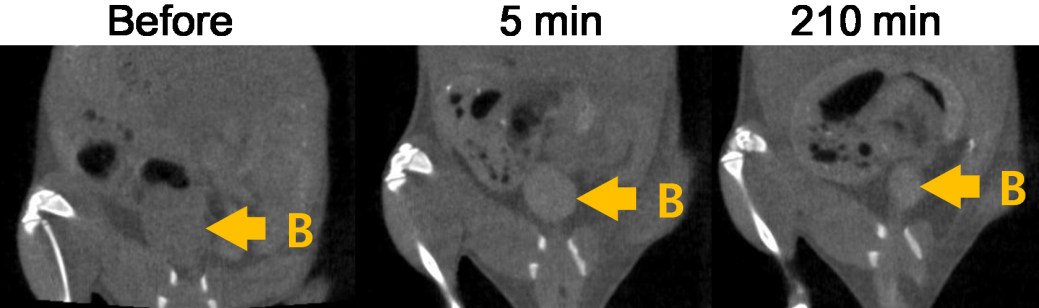


**Figure S7.** In vivo CT images of a mouse at an X-ray source voltage of 70 kV. B indicates the bladder.

**References**

1. Söderlind, F., Pedersen, H., Petoral Jr., R. M., Käll, P. -O. & Uvdal, K. Synthesis and characterization of Gd_2_O_3_ nanocrystals functionalized by organic acids. *J. Colloid Interface Sci*. **288**, 140-148 (2005).

2. Gd_2_O_3_, 1977 JCPDS-International Centre for Diffraction Data, card no. 43-1014, a = 10.813 Å.

3. Moulder, J. F., Stickle, W. F., Sobol, P. E. & Bomben, K. D. *Handbook of X Ray Photoelectron Spectroscopy*, Physical Electronics, **1995**.
